# Plasma and salivary hormone responses to a 30‐min exercise stress test in young, healthy, physically active females

**DOI:** 10.14814/phy2.70168

**Published:** 2024-12-25

**Authors:** Carla Baker, Jessica Piasecki, John A. Hunt, Gemma Foulds, John Hough

**Affiliations:** ^1^ SHAPE Research Centre, Department of Sport Science Nottingham Trent University Nottingham UK; ^2^ Medical Technologies Innovation Facility Nottingham Trent University Nottingham UK; ^3^ John Van‐Geest Cancer Research Centre Nottingham Trent University Nottingham UK

**Keywords:** biomarker, female, hormone, intensified training, overreaching

## Abstract

Overreaching, a consequence of intensified training, is used by athletes to enhance performance. A blunted hormonal response to a 30‐min interval exercise stress test (55/80) has been shown in males after intensified training, highlighting cortisol and testosterone as potential biomarkers of overreaching. Despite accounting for ~50% of the population, studies into hormonal responses to exercise in females are lacking. The menstrual cycle and oral contraceptives profoundly affect hormonal responses, necessitating separate investigations into the female response to the same exercise‐stress test. On three separate visits, 13 females (6 oral contraceptive users, 7 eumenorrheic) completed a VO_2max_ test, resting control trial, and 55/80 stress test. The 55/80 involves alternating between 1 min at 55% VO_2max_ and 4 min at 80% VO_2max_. Blood and saliva were collected pre, post, and 30 min post‐55/80, and at coinciding time points during the resting control trial. Plasma progesterone, estrogen, and plasma and salivary cortisol and testosterone were analyzed via ELISA. A significant elevation of salivary and plasma cortisol (~141% and ~87%, respectively, *p* < 0.001), salivary testosterone (~93%, *p* < 0.001), and plasma progesterone (~58%, *p* = 0.004) were evident from pre‐ to post‐55/80. Plasma testosterone remained unchanged. Hormonal responses were attenuated in oral contraceptive users. The 55/80 induces hormonal elevations in females, similar in magnitude as males.

## 1 INTRODUCTION

Overreaching is often used by athletes during a typical training cycle to enhance performance (Meeusen et al., 2013). Whilst initially the intensified training can result in a decline in performance, when appropriate periods of recovery are implemented, a super‐compensatory effect resulting in enhanced performance above baseline levels can occur (Meeusen et al., 2013). However, if the stress/recovery balance is not carefully monitored, non‐functional overreaching (NFOR) which can last weeks to months, or more severely, the overtraining syndrome (OTS) can occur (Meeusen et al., 2013). The OTS is surprisingly not infrequent amongst the athletic population and can take months to years for full recovery to occur (Meeusen et al., 2013). Previously reported symptoms of NFOR/OTS include poor sleep quality, increased illness incidence, a decline in performance, and low moods or depression to name a few (Weakley et al., 2022; Witard et al., 2014). Despite the high prevalence of the OTS amongst those with highly physical occupations, that is, athletes and military personnel, there are currently no clear biomarkers to identify its occurrence, and it is often retrospectively diagnosed, prolonging recovery time (Tanskanen et al., 2011; Weakley et al., 2022).

Previous groups have identified hormones associated with the hypothalamus and pituitary glands, such as cortisol and testosterone as possible biomarkers of the OTS (Hough et al., 2021; Meeusen et al., 2013). Cortisol is a glucocorticoid hormone secreted by the adrenal cortex in response to stresses, such as heavy exercise (McMurray & Hackney, 2000). Stress causes the hypothalamus to produce corticotropin‐releasing hormone, which stimulates the release of adrenocorticotropic hormone from the anterior pituitary gland, and in turn, the release of cortisol from the adrenal cortex; coined the hypothalamic–pituitary–adrenal axis (HPA axis) (Viru & Viru, 2004). The hypothalamic–pituitary‐gonadal axis (HPG axis) is responsible for the production of testosterone and progesterone, thus, both hormones follow a similar pathway of synthesis. The hypothalamus releases gonadotropin‐releasing hormone which stimulates the secretion of the gonadotropins; luteinising hormone and follicle‐stimulating hormone from the pituitary gland, which in turn stimulates the production of testosterone from the testes in males and ovaries in females (Sharma et al., 2022). In females, the majority of testosterone is produced by the ovaries (25%–50%) and adrenal glands (25%), with the remaining testosterone synthesized by the conversion of androstenedione to testosterone (Parish et al., 2021). Although limited differences between “healthy” and overtrained athletes occur at rest, studies have shown that in response to a stress stimulus, cortisol and testosterone are blunted (Hough et al., 2013, 2015; Meeusen et al., 2010). It is therefore suggested that hormone levels behave more homogenously upon stimulation compared to an “at rest” measure between “healthy” and overtrained cohorts (Carrard et al., 2022).

Regarding the development of a laboratory protocol required for the study of the OTS, Meeusen et al. (2004) developed an exercise stress protocol consisting of two maximal cycling exercise bouts separated by a 4‐h resting recovery. They reported a ~118% reduction in exercise‐induced plasma cortisol concentrations to the second maximal exercise bout after a 10‐day period where training volume was increased by 58%. However, the two‐exercise bout protocol and lengthy recovery time used may make this an impractical tool for athletes. Hough et al. (2011) therefore developed a 30‐min high‐intensity cycling protocol, the 55/80, consisting of 1 min at 55% VO_2max_ and 4 min at 80% VO_2max_. This stress test induced robust elevations in both salivary and plasma cortisol (~210% and ~91% from pre to post 55/80, respectively) and salivary testosterone (~58% pre to post 55/80) in healthy males (Hough et al., 2011). When implemented before and after an 11‐day intensified training period, the 55/80 highlighted a 166% (cortisol) and 21% (testosterone) reduction in the peak salivary hormonal responses (Hough et al., 2013). These maladaptive hormonal findings were coupled with increased fatigue and burnout scores reported via the psychological stress and recovery questionnaire (REST‐Q), indicating a possible state of NFOR/OTS (Hough et al., 2013, 2015).

To date, research in the field of overreaching/overtraining took the path of least confounding variables, placing an emphasis on male physiology. All of the aforementioned studies investigating the usefulness of exercise stress tests as a tool to highlight the OTS utilized either physically active males or elite male athletes (Hough et al., 2013). A recent review has established that female athletes are underrepresented in overtraining studies, likely due to complications associated with the biological complexity of the menstrual cycle (Carrard et al., 2022). Albeit female inclusion makes the science more complicated, that leaves ~50% of the population for which the necessary is still missing. We know that both the natural menstrual cycle phases, and oral contraceptive use impacts circulating cortisol concentrations (Hertel et al., 2017). Specifically, in oral contraceptive users, the oral contraceptives have been shown to elevate circulating cortisol levels (Hertel et al., 2017). Additionally, in naturally menstruating (eumenorrheic) females, cortisol increases in response to mental stress activation of the HPA axis are greater in the luteal phase (where progesterone is high) compared to other phases (Montero‐López et al., 2018). It is therefore not appropriate to assume that the findings shown in previous studies utilizing male participants can be directly applied to female athletes. Matos et al. (2011) identified that of 376 screened young English athletes, the incidence of self‐reported non‐functional overreaching or the OTS was significantly higher in females than males, highlighting the importance of including females into future investigations, even more so given the increased female participation in sport in general. As such, to develop female‐inclusive tools in the identification of the OTS, it is first necessary to assess whether the 55/80 can also induce robust elevations in cortisol and testosterone levels in females.

Therefore, the current study aimed to establish the salivary and plasma cortisol and testosterone responses in young, healthy, physically active females to the 55/80 to assess its usefulness as a tool in the diagnosis of the overtraining syndrome in females. Additionally, as the main female sex hormone and precursor to testosterone, we will also investigate the 55/80‐induced progesterone changes to equally establish its usefulness.

## 2 MATERIALS AND METHODS

### 2.1 Participants

Thirteen healthy, physically active, non‐smoking females volunteered to take part in this study. Six participants were oral contraceptive users taking a combined oral contraceptive pill (for at least 6 months prior to the study). The type of oral contraceptive pill was regulated (150 mg/30 mg, Levonorgestrel/Ethinylestradiol). Seven participants were eumenorrheic (natural menstruators) and had not used oral contraceptives for a minimum of 6 months prior to the study and must have had at least nine menstrual cycles in the past 12 months. The participant characteristics are outlined in Table 1. The study was approved by the Nottingham Trent University Invasive Ethics Committee (Ethics approval #573). After providing a detailed verbal and written explanation of the study, written informed consent and health screen were obtained from each participant prior to testing.

**TABLE 1:** Descriptive characteristics of participants.

	Group	Oral contraceptive users	Natural menstruators
Age (y)	26 ± 4	24 ± 5	28 ± 2
Height (cm)	163.1 ± 11.8	157.3 ± 12.2	168.0 ± 8.9
Weight (kg)	62.0 ± 8.5	57.6 ± 3.3	65.8 ± 9.6
BMI (kg/m^2^)	23.6 ± 3.8	23.8 ± 4.8	23.4 ± 2.6
VO_2max_ (mL.kg^−1^.min^−1^)	40.95 ± 5.68	39.97 ± 4.33	41.93 ± 6.62

*Note*: *n* = 13. Data are presented as mean ± SD.

### 2.2 Experimental design

**FIGURE 1:**
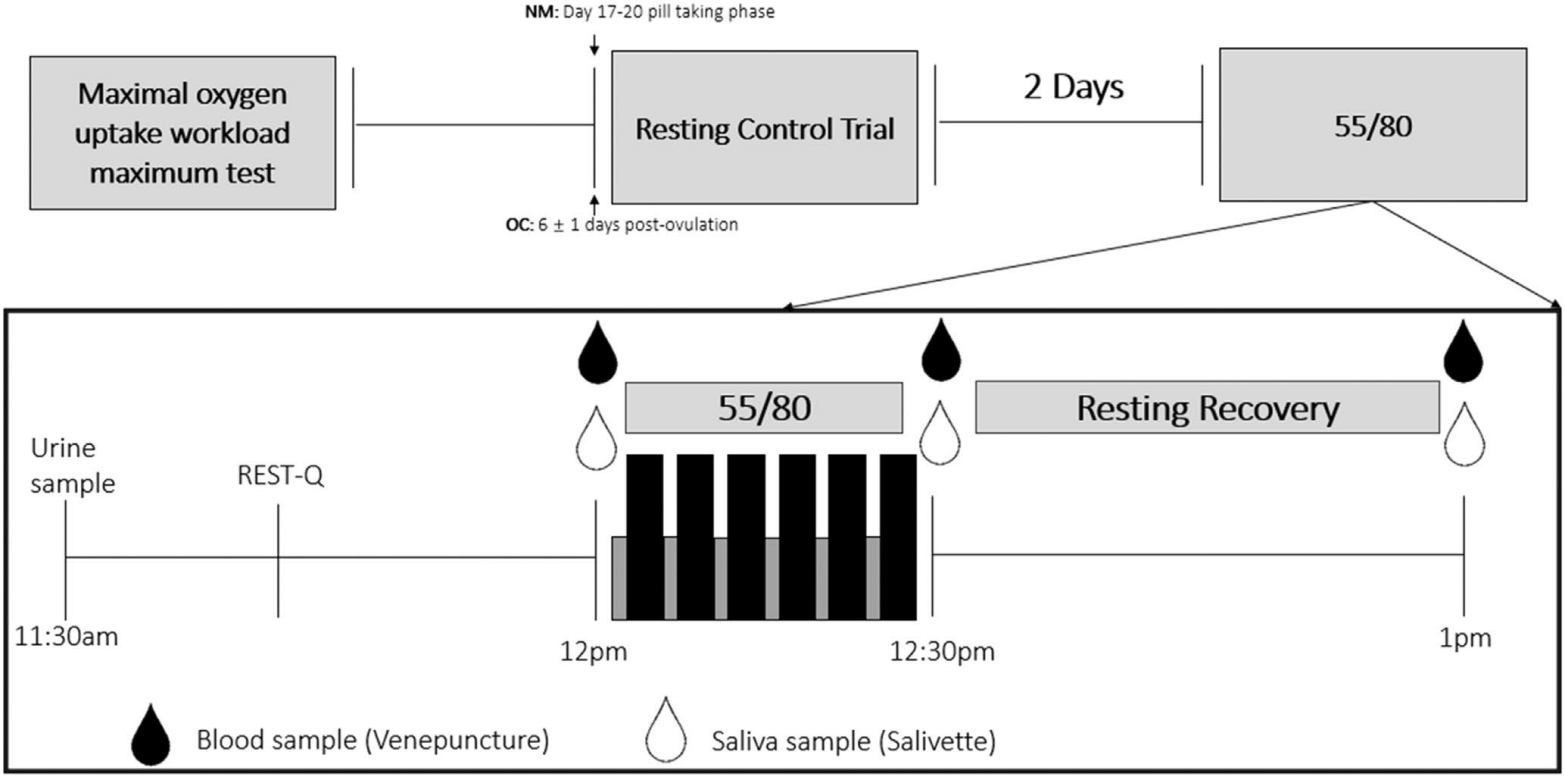
Schematic representing the overall experimental design and specifics of the 55/80 trial. The resting control trial was the same as the 55/80 trial, but participants completed seated rest instead of the 55/80 cycle bout.

This study was a non‐randomized, repeated measures‐controlled trial. Each participant completed three separate visits to the laboratory: one session for preliminary measures and two main experimental trials (Figure 1). To avoid any effect of circadian rhythm, the two main experimental trials (visit 2 and 3) occurred at the same time of day. Participants were instructed to abstain from exercise, caffeine, and alcohol 24 h prior to testing. Both main experimental trials were completed within 3 weeks of visit 1 and 2 days apart from each other. On the main experimental trial days, participants were instructed to consume a standardized breakfast at the same time of day and drink at least 500 mL of water on the morning before each visit to ensure hydration, as hypohydration can lead to artificially elevated hormone levels. Once in the laboratory, urine osmolality was assessed with an osmolality of <700 mOsmol.kg^−1^ H_2_O being acceptable for blood sampling. If participants did not meet these criteria, they were instructed to consume 500 mL of water and wait for 10 more minutes before being retested.

### 2.3 Menstrual cycle control

Both main trial days occurred within the luteal phase of the menstrual cycle. This was identified as being between 6 ± 1 days post‐ovulation (main experimental trial 1) and 9 ± 1 days post‐ovulation (main experimental trial 2) for natural menstruators (*n* = 7). Participants were given daily ovulation status urinary kits to use following the final day bleed to confirm ovulation (Clearblue®, Bedford, UK). Oral contraceptive users (*n* = 6) completed their first main experimental trial in the middle of the pill‐taking phase (day 17–20 following beginning of withdrawal bleed). Phase verification was confirmed via blood analysis of progesterone and estrogen.

### 2.4 Preliminary measures

Height (Seca 217 stadiometer, Seca, Hamburg, Germany) and body mass (Seca 761 scales, Seca, Hamburg, Germany) were collected using standard methods, and cardiopulmonary fitness (VO_2max_) was assessed on a Lode Excalibur Sport electronically braked cycle ergometer (Lode, Groningen, Holland), using a continuous step protocol, starting at 60 W increasing by 35 W every 3 min until volitational fatigue. Expired air was assessed throughout the test for oxygen consumption and carbon dioxide production using breath‐by‐breath analysis (Metalyzer 3B, Cortex Medical, Germany) for VO_2peak_ to be calculated. Heart rate (HR) was assessed using a heart rate monitor (Polar F2, Polar Electro Oy, Kempele, Finland) and ratings of perceived exertion (RPE) recorded using the Borg scale (Borg, 1982). Maximum power output (Wmax) was determined using the equation W_max_ = W_final_ + (*t*/*T*)W_inc_ (Kreider et al., 1998), where W_final_ is the power output during the final stage completed, *t* is the amount of time (seconds) reached in the final uncompleted stage, *T* is the duration of each stage (180 s), and W_inc_ is the workload increment (35 W). Power outputs equivalent to 55% and 80% were calculated for each participant for use in their main trials.

### 2.5 Main trials

#### 2.5.1 Main trial 1: Resting control

7–21 days after preliminary testing, participants reported to the lab for their first main experimental trial at 11:30 am. Participants undertook seated rest whilst completing the 76‐item Recovery‐Stress Questionnaire for Athletes (RESTQ‐76 Sport) which measures stress and recovery levels over the last 3‐days (Kellmann and Kallus, 2001). A similar difference between total stress and total recovery scores obtained by the REST‐Q indicates participants were not more or less stressed/recovered before completing each experimental trial. 12 pm, 4–6 mL blood was collected via venepuncture from the forearm into one EDTA vacutainer, and a saliva sample was collected via a Salivette (Sarstedt, Leicester, UK). This was repeated at 12:30 pm and 1 pm. Participants were allowed water ad libitum but not during the 10 min prior to saliva sampling to avoid the possibility of saliva sample dilution.

#### 2.5.2 Main trial 2: The 55/80

Two days after the completion of the resting control, participants completed their second and final main experimental trial. All procedures were the same as during the resting control, but between 12:30 pm and 1 pm, participants underwent a 30‐min cycle consisting of alternating blocks of 1 min at 55% VO_2max_ and 4 minutes at 80% VO_2max_. HR and RPE were collected at the end of each stage.

#### 2.5.3 Blood and saliva analysis

All blood and saliva samples were immediately centrifuged at 2000 ***g*** at 4°C for 15 min, and aliquoted into 1.5 mL cryovial tubes, frozen and stored at −80°C until analysis. Plasma cortisol (R&D systems, Minneapolis, USA. Catalog #KGE008B), testosterone (DRG Instruments, Marburg, Germany. Catalog #EIA1559), progesterone (catalog #ab108670)and estrogen (catalog #ab285239) (Abcam, Cambridge, UK), as well as salivary cortisol (catalog #1–3002) and testosterone (catalog #1–2402) (Salimetrics, Pennsylvania, USA) were analyzed using commercially available enzyme‐linked immunosorbent assay (ELISA) kits. The mean intra‐assay coefficient of variation (CV) for all ELISA plates was <10.4%, and inter‐assay <13.4%, apart from the plasma cortisol plate which was <13.2% and <17.5%, respectively.

### 2.6 Statistical analyses

Data were examined using the SPSS statistical package version 28 (IBM Corporation, Armonk NY USA) for normal distribution using the Kolmogorov–Smirnov test. Non‐normally distributed data were logarithmically transformed and re‐examined. A two‐way repeated measures analyses of variance (ANOVA) with a Bonferroni correction was used to examine the effects of trial (Resting control vs. 55/80), time and a Trial*Time interaction on the cortisol, testosterone, and progesterone concentrations. When the assumption of sphericity was violated, a Greenhouse–Geisser correction was applied. Paired samples *t*‐tests were used for post‐hoc analysis and to examine differences between plasma estrogen and progesterone at rest and REST‐Q questionnaire scores between trials. Statistical significance was accepted at the *p* < 0.05 level. Data are presented as mean ± standard deviation (SD). One participant was excluded from progesterone analysis due to anomalies in the data, and two were excluded from estrogen analysis due to missed blood samples.

### 2.7 Justification of sample size

Sample size estimations were based on our primary outcome variables, cortisol and testosterone. Completing an a priori power calculation, it was estimated that 12 participants would be required to see a meaningful difference in the main outcome variables in response to the exercise stress test (Cohen's *d* = 0.90) at 80% power and an *α* level of 0.05. To account for a 10% drop‐out rate, 13 participants were recruited.

## 3 RESULTS

### 3.1 Confirmation of menstrual cycle phase

The plasma progesterone to estrogen ratio at rest during the control and 55/80 trials were 1.94 and 3.34 ng/mL, respectively, confirming that testing took place during the early to mid‐Luteal phase (Table 2). There were no significant differences in mean resting plasma estrogen (*t*(9) = 0.188, *p* = 0.855) or progesterone concentrations between the 55/80 and resting control trials (*t*(10) = −1.132, *p* = 0.284) (Table 2).

**TABLE 2:** Resting plasma progesterone to estrogen ratio (*n* = 11), plasma progesterone (*n* = 11), and plasma Estrogen (*n* = 10) during the resting control and 55/80 trial visits.

	Resting control	55/80
Plasma Progesterone: Estrogen Ratio (ng/mL)	1.94 (0.81)	3.34 (1.87)

	Group mean	Oral contraceptive users	Natural menstruators	Group mean	Oral contraceptive users	Natural menstruators
Plasma Progesterone (ng/mL)	1.37 (1.39)	0.68 (0.29)	5.69 (7.89)	2.29 (3.63)	0.68 (0.34)	10.28 (12.59)
Plasma Estrogen (pg/mL)	705.90 (179.21)	604.47 (76.49)	807.33 (194.44)	685.89 (109.92)	663.51 (133.28)	708.27 (73.50)

*Note*: Data are presented as mean (standard deviation).

### 3.2 55/80 HR, RPE, and %Wmax

The mean heart rate during the 55/80 trial was 165 ± 9 bpm, and the mean RPE was 15 ± 1. The average 55% maximum power output was 108 ± 18 W, and the mean 80% maximum power output was 156 ± 27 W.

### 3.3 Rest‐q

There was no significant differences in the total recovery – total stress REST‐Q scores between trials (*t*(9) = −0.043, *p* = 0.967). Specifically, the total recovery and total stress were similar during the 55/80 trial (1.75 ± 1.02) and the control trial (1.74 ± 0.91).

### 3.4 Salivary cortisol

**FIGURE 2:**
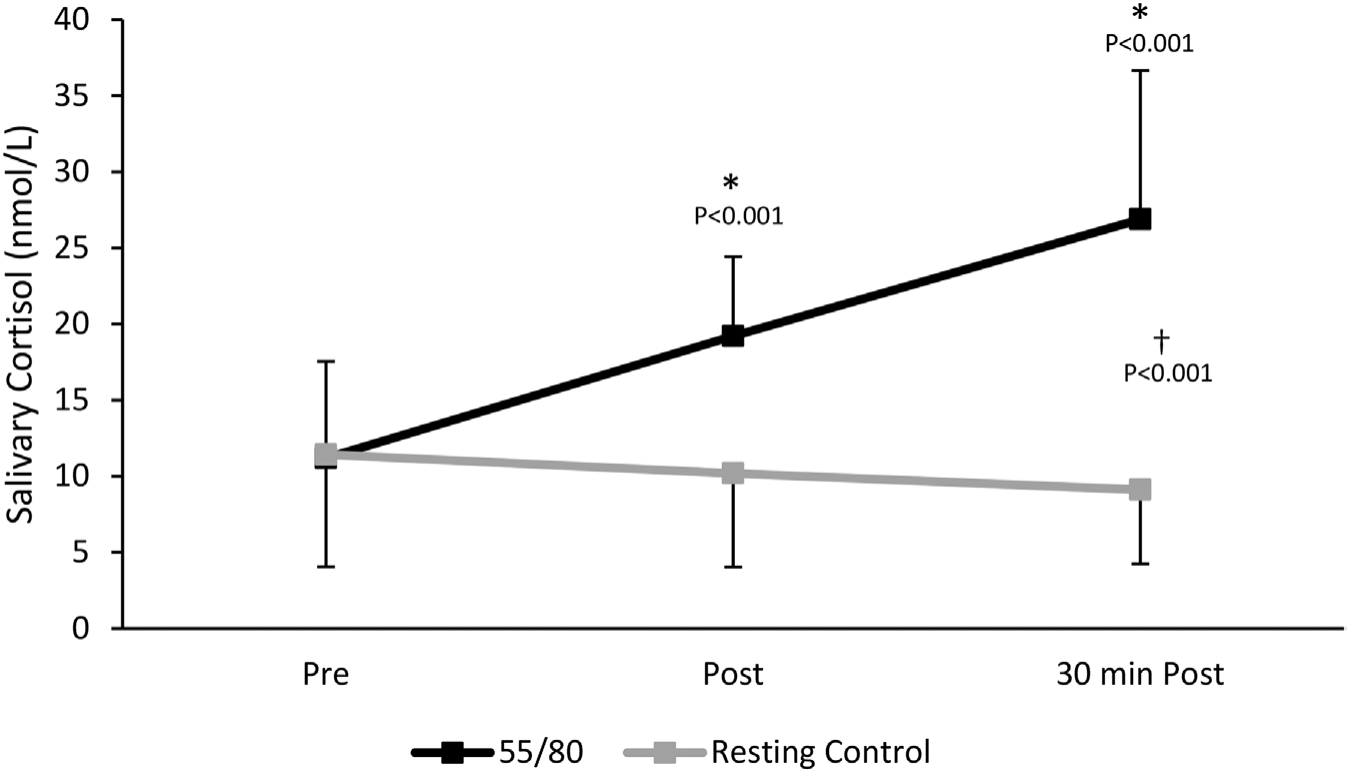
Salivary cortisol concentrations during the resting control trial and the 55/80 trial. Data are presented as mean ± standard deviation. *Significantly different from baseline. ^†^Significant effect of condition. *n* = 13.

There was a significant main effect of trial (*F*(1,12) = 38.873, *p* < 0.001) whereby mean salivary cortisol levels were higher in the 55/80 trial compared to the resting control trial (19.09 ± 1.65 vs. 10.24 ± 1.71 nmol/L, *p* < 0.001). Time was also a significant main effect wherein salivary cortisol levels were elevated in response to the 55/80 exercise bout (*F*(2,24) = 11.009, *p* = 0.004) (Figure 2). Post hoc analysis revealed that salivary cortisol was elevated above baseline (11.18 ± 6.64 mmol/L) immediately post (19.20 ± 5.44 mmol/L) and 30 min post (26.89 ± 10.18) 55/80 (*p* < 0.001). The significant interaction between time point and trial (*F*(2,24) = 36.335, *p* < 0.001) indicates that the change in salivary cortisol across the various timepoints differed between the 55/80 trial and the resting control trial.

**FIGURE 3:**
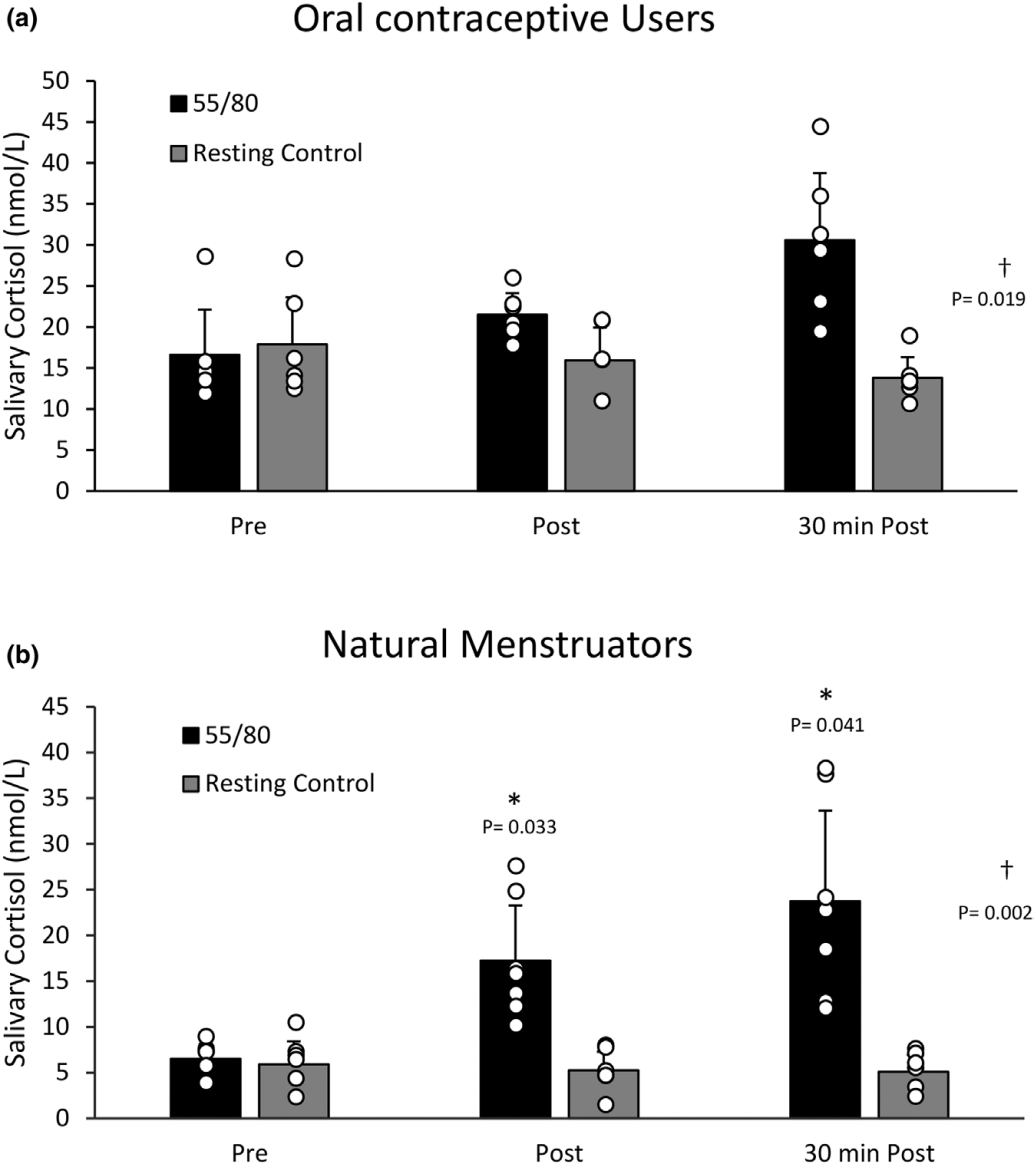
(a) Salivary cortisol concentrations during the resting control trial and the 55/80 trial in oral contraceptive users (*n* = 6). Data are presented as mean ± standard deviation. *Significantly different from baseline. †Significant effect of condition. (b) Salivary cortisol concentrations during the resting control trial and the 55/80 trial in natural menstruators (*n* = 7). Data are presented as mean ± standard deviation. *Significantly different from baseline. †Significant effect of condition.

When oral contraceptive users (*n* = 6) and natural menstruators (*n* = 7) were compared, natural menstruators demonstrated a larger post‐exercise (~165%) and 30 min post‐exercise (~265%) increase in salivary cortisol compared to oral contraceptive users (~30% and ~84%, respectively) from baseline (Figure 3a). For natural menstruators, there was a significant main effect of trial (*F*(1,6) = 30.188, *p* = 0.002) whereby mean salivary cortisol levels were higher in the 55/80 trial (15.82 ± 5.99 nmol/L) than the control trial (5.41 ± 1.83 nmol/L), time (*F*(1.053, 6.318) = 11.752, *p* = 0.012), and time*trial interaction (*F*(2, 12) = 31.494, *p* < 0.001) (Figure 3b). Post hoc analysis revealed that salivary cortisol was elevated above baseline (6.52 ± 5.90 mmol/L) immediately post (17.23 ± 5.27 mmol/L, *p* = 0.033) and 30 min post (23.72 ± 5.08, *p* = 0.041) 55/80. Within the oral contraceptive users, there was a significant main effect of trial (*F*(1,5) = 11.710, *p* = 0.019), whereby mean salivary cortisol levels were higher in the 55/80 trial (22.90 ± 3.15 nmol/L) compared to the control trial (15.87 ± 4.11 nmol/L) but no main effect of time (*F*(1.099,5.495) = 2.274, *p* = 0.188) on salivary cortisol concentrations.

### 3.5 Salivary testosterone

**FIGURE 4:**
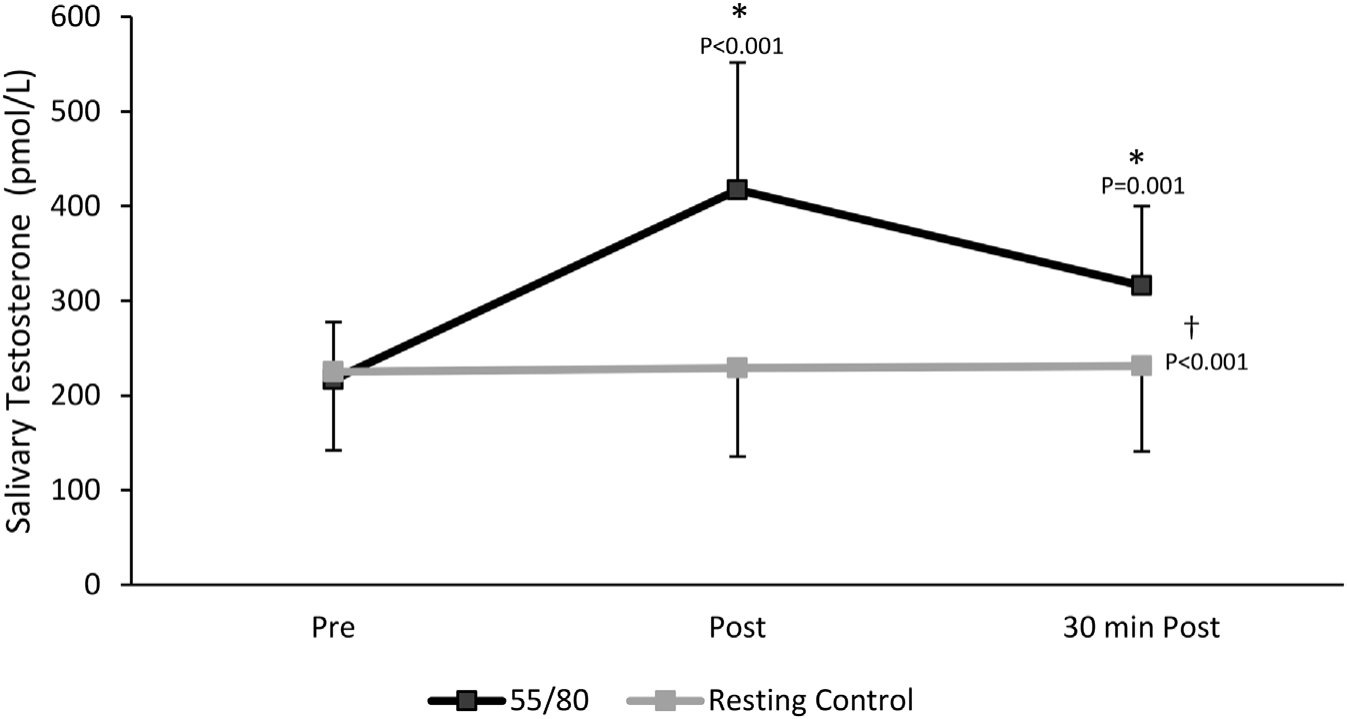
Salivary testosterone concentrations during the resting control trial and the 55/80 trial. Data are presented as mean ± standard deviation. *Significantly different from baseline. †Significant effect of condition. *N* = 13.

There was a significant main effect of trial (*F*(1,12) = 151.216, *p* < 0.001), whereby mean salivary testosterone levels were higher in the 55/80 trial compared to the resting control trial (316.59 ± 127.77 vs. 228.22 ± 88.96 pmol/L, *p* < 0.001). There was also a significant main effect of time whereby salivary testosterone levels were elevated in response to the 55/80 exercise bout (*F*(2,24) = 9.680, *p* < 0.001) (Figure 4). Post‐hoc analysis revealed that salivary testosterone was significantly elevated above baseline (216.55 ± 63.39 pmol/L) immediately post (417.23 ± 140.03 pmol/L, *p* < 0.001) and 30 minutes post 55/80 (315.99 ± 87.25 pmol/L, *p* = 0.001). The significant interaction between time point and trial (*F*(2,24) = 68.754, *p* < 0.001) indicates that the change in salivary testosterone across the various timepoints differed between the 55/80 trial and the resting control trial.

**FIGURE 5:**
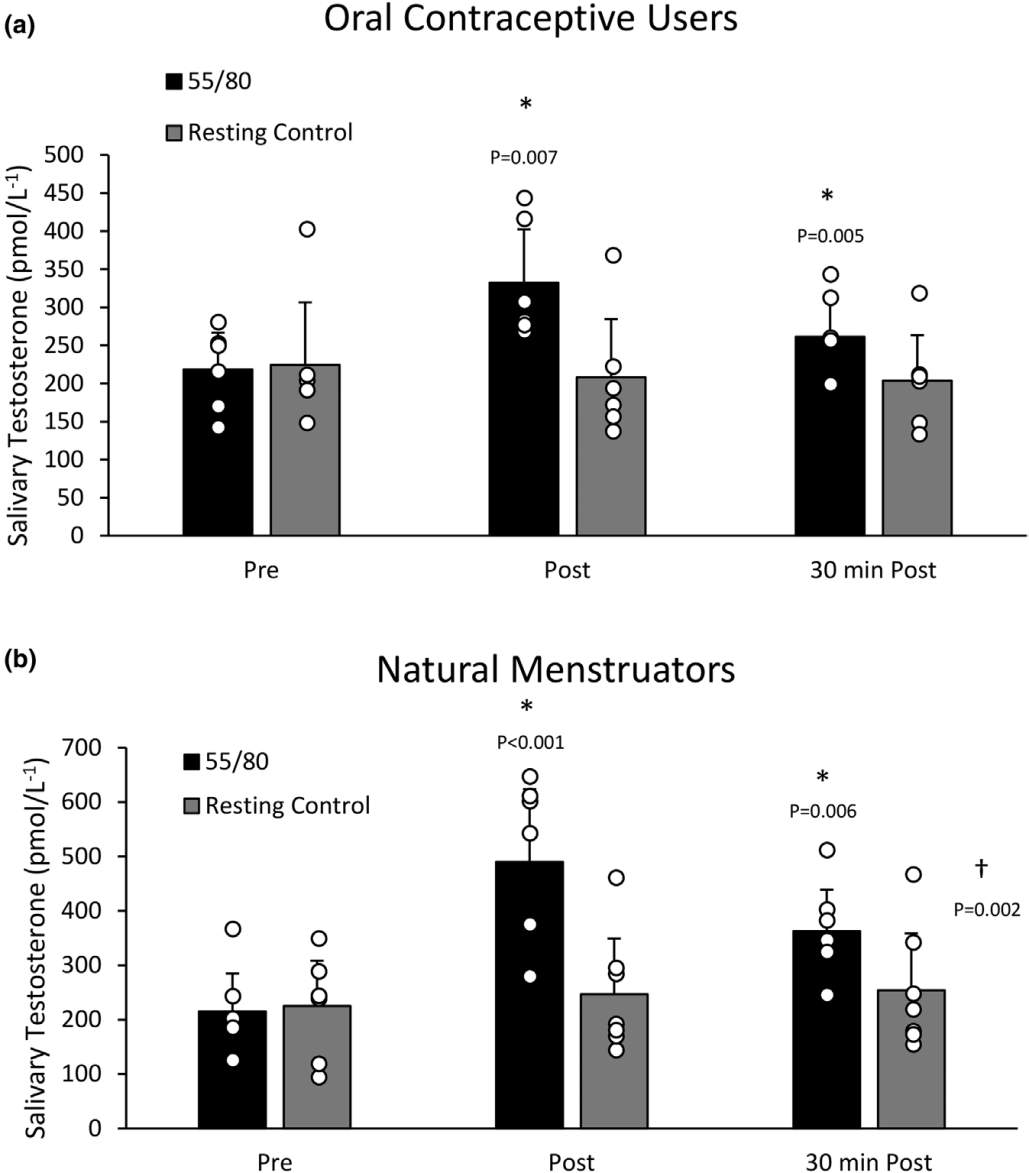
(a) Salivary testosterone concentrations during the resting control trial and the 55/80 trial in oral contraceptive users (*n* = 6). Data are presented as mean ± standard deviation. *Significantly different from baseline. †Significant effect of condition. (b) Salivary testosterone concentrations during the resting control trial and the 55/80 trial in natural menstruators (*n* = 7). Data are presented as mean ± standard deviation. *Significantly different from baseline. †Significant effect of condition.

Within the oral contraceptive users, there was no significant main effect of trial (*F*(1,5) = 5.289, *p* = 0.070), but there was a significant main effect of time (*F*(2, 10) = 5.208, *p* = 0.028) and time*trial interaction (F(2, 10) = 25.522, *p* < 0.001). Post‐hoc analysis revealed that salivary testosterone was significantly elevated above baseline (218.33 ± 53.19 pmol/L) immediately post (332.14 ± 77.16 pmol/L, *p* = 0.007) and 30 min post 55/80 (261.33 ± 58.79 pmol/L, *p* = 0.005) (Figure 5a). For natural menstruators, there was a significant main effect of trial (*F*(1,6) = 27.789, *p* = 0.002), whereby mean salivary testosterone levels were higher in the 55/80 trial (356.01 ± 85.78 pmol/L) compared to the control trial (241.97 ± 83.68 pmol/L), time (*F*(2,12) = 2.274, *p* < 0.001), and time*trial interaction (*F*(2,12) = 8.325, *p* = 0.005) (Figure 5b). Post‐hoc analysis revealed that salivary testosterone was significantly elevated above baseline (215.03 ± 75.31 pmol/L) immediately post (490.17 ± 144.25, *p* < 0.001) and 30 min post 55/80 (362.85 ± 82.43, *p* = 0.006). When oral contraceptive users (*n* = 6) and natural menstruators (*n* = 7) were compared, natural menstruators demonstrated a larger post‐exercise (~128%) and 30 min post‐exercise (~68%) increase in salivary testosterone compared to oral contraceptive users (~52% and ~20%, respectively) from baseline.

### 3.6 Plasma testosterone

**FIGURE 6:**
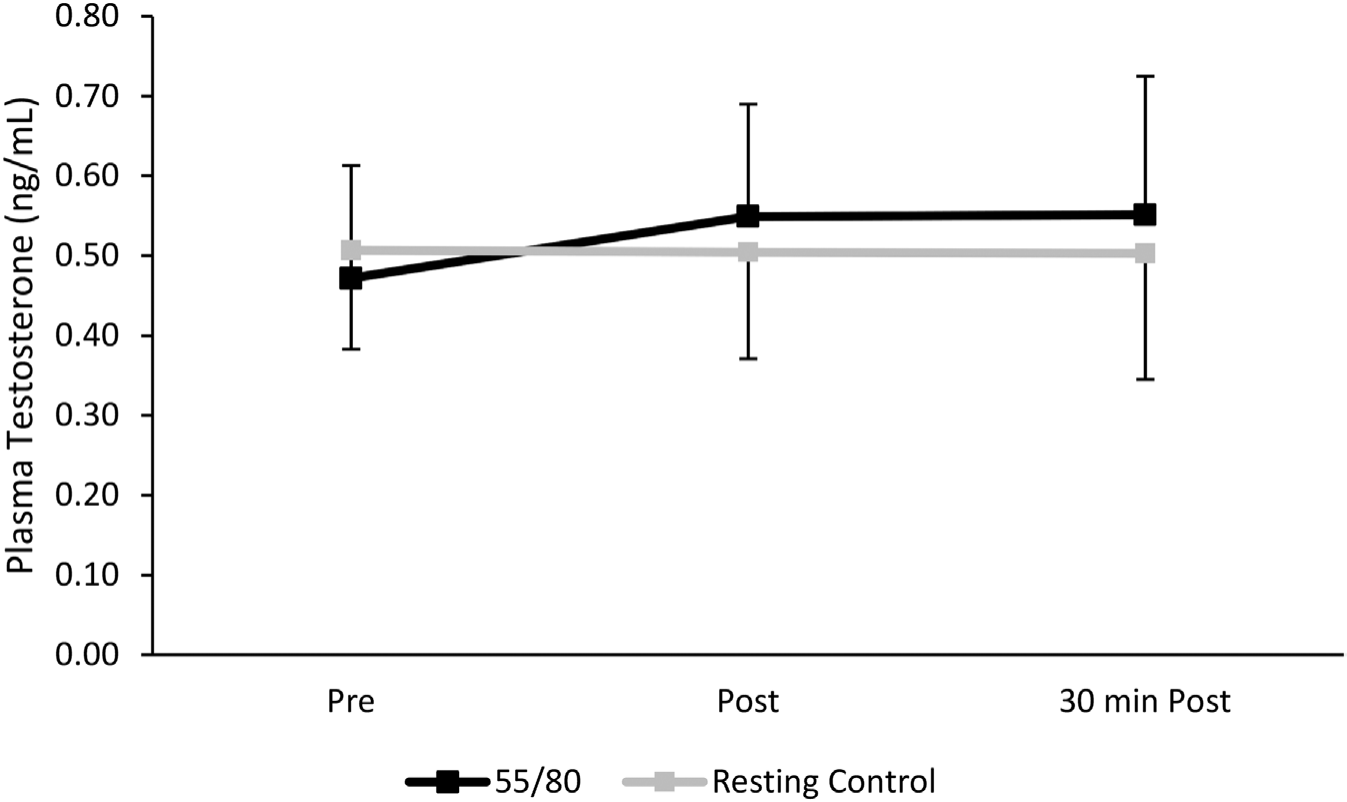
Plasma testosterone concentrations during the resting control trial and the 55/80 trial.

There were no significant differences in plasma testosterone between trials (*F*(1,11) = 0.646, *p* = 0.439) or time (*F*(2,22) = 0.1586, *p* = 0.227) (Figure 6).

### 3.7 Plasma cortisol

**FIGURE 7:**
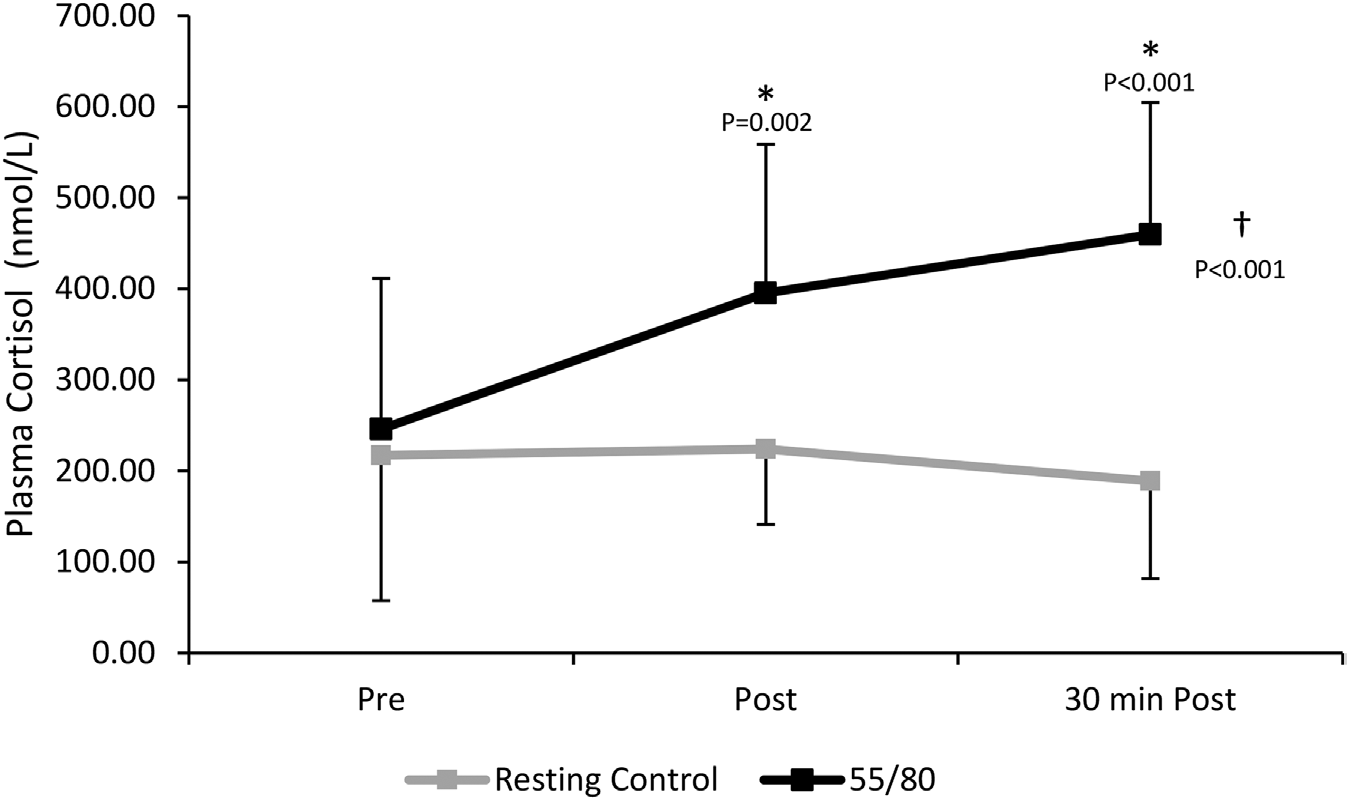
Plasma cortisol concentrations during the resting control trial and the 55/80 trial. Data are presented as mean ± standard deviation. *Significantly different from baseline. †Significant effect of condition. *N* = 12.

There was a significant main effect of trial (*F*(1,11) = 20.855, *p* < 0.001), whereby mean plasma cortisol levels were higher in the 55/80 trial compared to the resting control trial (367.01 ± 146.37 vs. 210.07 ± 152.23 nmol/L, *p* < 0.001) (Figure 7). There was also a significant main effect of time whereby plasma cortisol levels elevated in response to the 55/80 exercise bout (*F*(2,22) = 12.553, *p* < 0.001). Post‐hoc analysis revealed that plasma cortisol levels were significantly elevated above baseline (246.606 ± 159.71 nmol/L) both immediately post (395.32 ± 82.86 nmol/L, *p* = 0.002) and 30 minutes post 55/80 (459.65 ± 107.45 nmol/L, *p* < 0.001). The significant interaction between time point and trial (*F*(2,11.964) = 20.540, *p* < 0.001) indicates that the change in plasma cortisol across the various timepoints differed between the 55/80 trial and the resting control trial.

**FIGURE 8:**
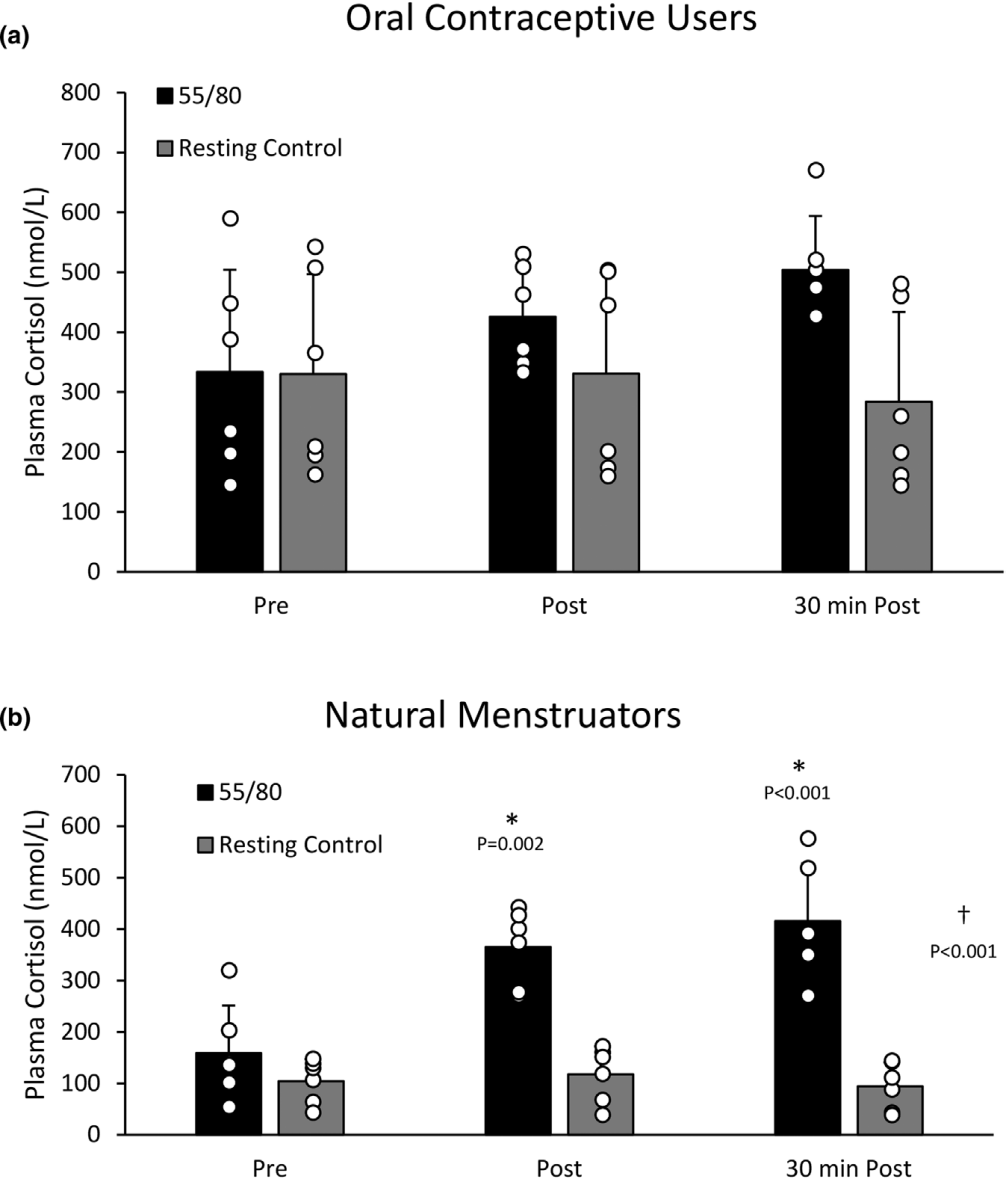
(a) Plasma cortisol concentrations during the resting control trial and the 55/80 trial in oral contraceptive users (*n* = 6). Data are presented as mean ± standard deviation. (b) Plasma cortisol concentrations during the resting control trial and the 55/80 trial in natural menstruators (*n* = 6). Data are presented as mean ± standard deviation. *Significantly different from baseline. †Significant effect of condition.

Within the oral contraceptive users, there was no significant effect of trial (*F*(1,5) = 6.065, *p* = 0.057) or time (*F*(2,10) = 1.294, *p* = 0.316) on plasma cortisol concentrations (Figure 8a). For natural menstruators, there was a significant main effect of trial (*F*(1,5) = 57.652, *p* < 0.001), whereby mean plasma cortisol levels were higher after the 55/80 trial (420.98 ± 131.28 nmol/L) compared to the control trial (314.86 ± 149.57 nmol/L), time (*F*(2,10) = 24.965, *p* < 0.001), and time*trial interaction (*F*(2,10) = 31.866, *p* < 0.001) (Figure 8b). Post‐hoc analysis revealed that plasma cortisol was significantly elevated above baseline (158.54 ± 92.74 nmol/L) immediately post (365.00 ± 74.42 nmol.L, *p* = 0.002) and 30 min post 55/80 (415.59 ± 45.83 nmol/L, *p* < 0.001).

### 3.8 Plasma progesterone

**FIGURE 9:**
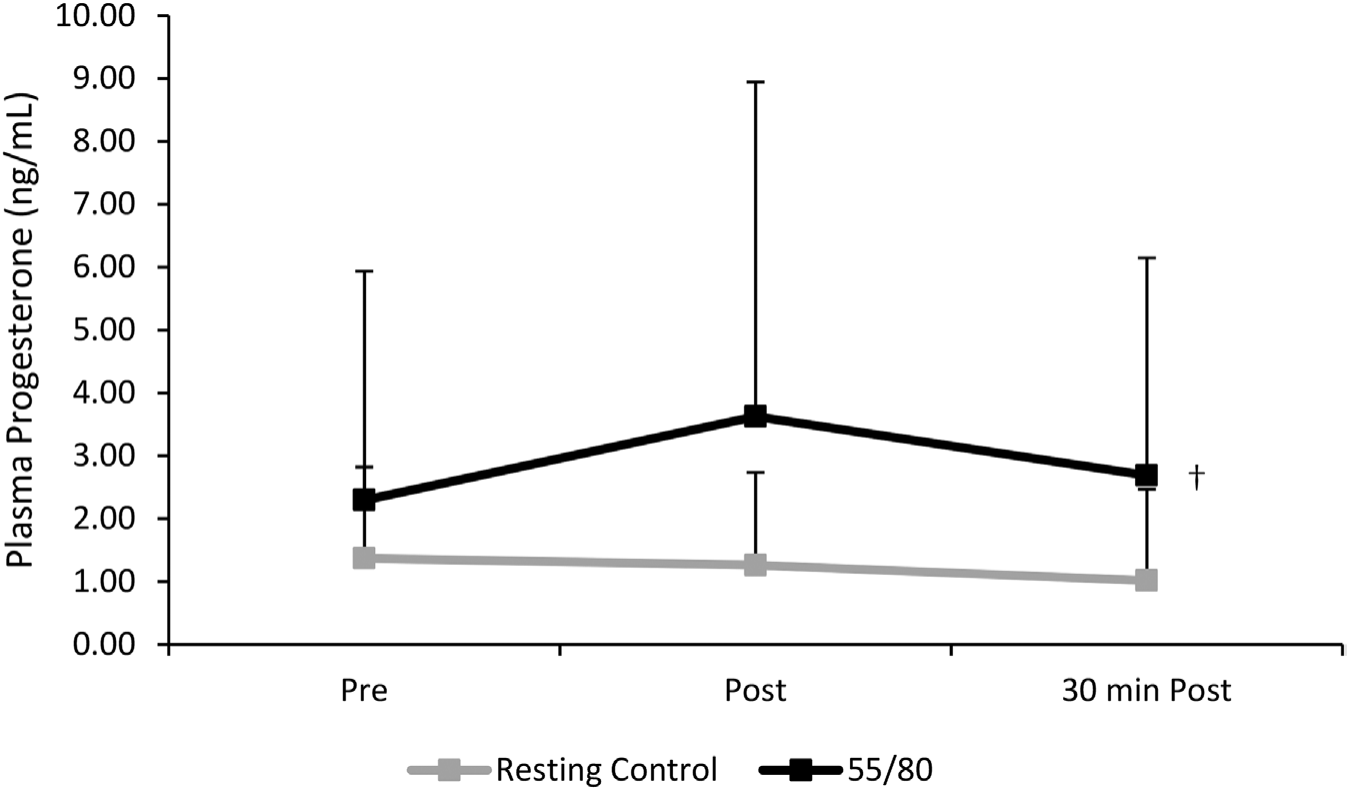
Plasma progesterone concentrations during the resting control trial and the 55/80 trial (*n* = 11). Data are presented as mean ± standard deviation. †Significant effect of condition.

There was a significant main effect of trial (*F*(1,10) = 14.083, *p* = 0.004), whereby mean plasma progesterone levels were higher in the 55/80 trial compared to the resting control trial (2.87 ± 0.56 vs. 1.22 ± 0.15 ng/mL). There was a non‐significant main effect of time (*F*(1.302,13.023) = 1.266, *p* = 0.295), but a significant time*trial interaction (*F*(1.146,11.458) = 4.910, *p* = 0.044), indicating that the change in plasma progesterone across the various timepoints differed between the 55/80 trial and the resting control trial (Figure 9).

## 4 DISCUSSION

The aim of this study was to examine whether the previously developed 55/80 stress test induced elevations in salivary and/or plasma cortisol and testosterone in females, as previously demonstrated in males, as well as in plasma progesterone. The goal was to determine and propose the test's usefulness as a diagnostic tool for overtraining syndrome in females. The current study experimentally evidenced that the 55/80 stress test induced significant elevations in salivary and plasma cortisol, salivary testosterone, and plasma progesterone in females. Plasma testosterone remained unchanged by the 55/80 exercise bout.

Specifically, the 55/80 elevations in salivary cortisol (~141%) and testosterone (~93%) from baseline to peak‐post exercise level were in line with those previously identified for the 55/80 in males, wherein both salivary cortisol and testosterone were elevated by ~210% and ~58%, respectively (Hough et al., 2013). Similarly, to the results of Hough et al. (2011) in males, we found a robust elevation in plasma cortisol (~87%), but no change in plasma testosterone in females. Our results do not align with all of the previously published research comparatively to studies using similar exercise durations and intensities, however. Duclos et al. (1997) found that 20 min of running exercise at either 50% or 80% VO_2max_ in highly trained males did not alter plasma cortisol levels. Training background was proposed as a possible cause for differences in cortisol perturbations to exercise (Wittert et al., 1996). It was argued that the stress intensity threshold required to provoke cortisol increases was typically higher in more highly trained individuals when comparing to those who are relatively sedentary (Hackney & Walz, 2013; Hill et al., 2008). The participants in the current study, and those used by Hough et al. (2011), were recreationally active with an average VO_2max_ classified as ‘Good’ according to the American College of Sports Medicine (ACSM, 2017). Duclos et al. (1997), although did not state the VO_2max_ of their participants, utilized highly trained long‐distance runners who ran 60–80 km/week for >4 years and were able to complete a marathon in <4 h. Therefore, differences in the training status of participants could explain why our study found plasma and salivary cortisol level increases, whereas other studies utilizing exercise of a similar intensity and duration may not have.

When the analysis of the hormone response to the 55/80 was conducted separately for oral contraceptive users and natural menstruators, there were no significant elevations in plasma cortisol or salivary testosterone in the oral contraceptive users, whereas salivary testosterone and cortisol, and plasma cortisol were significantly elevated in the natural menstruators. The natural menstruators had lower average resting salivary and plasma cortisol levels compared to the oral contraceptive users. It is already known that oral contraceptives elevate circulating cortisol levels in females by causing stress‐like alterations in the F056 binding protein FKBP5; a central regulator of the HPA axis (Hertel et al., 2017). This elevated baseline most likely explains why the oral contraceptive user group did not see significant elevations in their plasma cortisol levels to the 55/80. A ceiling effect of exercise‐induced plasma cortisol elevations has previously been shown in males (Behr et al., 2009). In this particular study, supra‐maximal exercise was used at varying intensities in male participants to identify a ceiling in plasma cortisol occurring at ~543–600 mmol/L (Behr et al., 2009). In the current study, the 55/80 elevated plasma cortisol to ~503 nmol/L in oral contraceptive users, nearing the ceiling level found in males. Therefore, starting with higher circulating plasma cortisol levels may have reduced the capacity for plasma cortisol elevation in the oral contraceptive users. Combined oral contraceptives have also been shown to reduce levels of androgen, especially testosterone, in females by inhibiting ovarian and adrenal androgen synthesis (Zimmerman et al., 2013). Therefore, the 55/80 may only induce significant plasma cortisol and salivary testosterone elevations in natural menstruators making it an unsuitable tool for highlighting the negative states of overtraining in oral contraceptive users. It must be emphasized, however, that although the overall study is sufficiently powered, when classifying participants as oral contraceptive users or natural menstruators, the analyses conducted are underpowered. Whilst the comparisons between oral contraceptive users and natural menstruators remain interesting, strong conclusions cannot be drawn.

In the current study, plasma testosterone was not significantly elevated by the 55/80. Hough et al. (2011) also found that the 55/80 failed to elevate plasma testosterone in males. Research has demonstrated that strength training elicits more pronounced elevations in circulating testosterone levels compared to aerobic exercise, attributed to the robust influence of the anaerobic glycolytic pathway in precipitating acute hormonal surges following physical exertion (Kraemer & Ratamess, 2005; Tremblay et al., 2004). It has also been shown that testosterone is more responsive to higher‐intensity exercise of longer durations (Cadore & Kruel, 2012). Consequently, the 55/80 protocol might lack the required potency to elicit increases in plasma testosterone levels. Additionally, a significant negative relationship, although low in magnitude, between circulating cortisol and total testosterone in males occurred in response to 60–90 min of either running, cycling, or rowing at ~65%–75% VO_2max_ (Brownlee et al., 2005). The authors proposed that a critical cortisol increase threshold of ~160% must be reached to substantially influence circulating testosterone levels; a threshold not reached in our research (Brownlee et al., 2005). Previous research in female runners also indicated that 30 min of running at a self‐selected pace, elevated plasma testosterone levels, and these were significantly greater when testing in the follicular phase compared to the luteal phase, despite baseline testosterone levels being similar in both phases (Shangold et al., 1981). The research presented from our study was taken from exercised female participants during the early luteal phase, which could have therefore contributed to the limited plasma testosterone responsiveness to the 55/80.

Plasma progesterone levels were significantly higher in the 55/80 trial compared to the resting control trial. As a secondary hypothesis, it was theorized that progesterone, as one of the two predominant female sex hormones and because it shares the same synthesis pathway as testosterone in females, may be altered in response to the 55/80 (Batth et al., 2020). Previous research has also found that 30 min of intense cycling is a strong enough stimulus to elevate serum progesterone levels in young healthy females (Bonen et al., 1979). Additionally, physical stress instilled via the cold pressor test, which requires participants to immerse their hand in ice‐cold water for 1–3 min, also led to elevations in salivary progesterone in young healthy females (Herrera et al., 2016). Similarly to testosterone as previously described, it is suggested that the progesterone response to stress is higher during the follicular phase of the menstrual cycle, rather than the luteal phase, thus, larger plasma progesterone elevations to the 55/80 may be seen if performed during the follicular phase (Herrera et al., 2016). Nevertheless, the significant elevations in plasma progesterone seen in the current study suggests it may be a more useful biomarker of the overtraining syndrome in females than plasma testosterone when testing is completed in the luteal phase.

Importantly, hormonal measurements were analyzed in euhydrated participants, meaning hydration status is unlikely to have influenced any hormonal changes. Additionally, the difference between total stress and total recovery measured by the REST‐Q scores were not significantly different between trials meaning the stress‐induced hormonal changes seen in the current study are likely due to the 55/80 exercise stress and not due to external stressors.

In conclusion, the results of this study support the use of the 55/80 as a potentially valuable tool, capable of highlighting differences in hormonal biomarkers associated with the negative states of overtraining in females. Specifically, plasma and salivary cortisol, salivary testosterone, and plasma progesterone were demonstrated to be indicative biomarkers of the overtraining syndrome in females. However, whilst inducing robust hormonal elevations in natural menstruators, oral contraceptive users demonstrated an attenuated response to the 55/80. The demonstration and useful utilization of this tool is important considering the high prevalence of the overtraining syndrome amongst the under‐researched and thus under‐represented female athlete population (Carrard et al., 2022; Matos et al., 2011). To improve the applicability of the 55/80 as a stress test, future research should investigate the hormonal perturbations in all phases of the menstrual cycle, that is, in the follicular phase, ovulatory period, and during menses to ensure a robust hormonal stress response remains. It is also vital that the 55/80 is validated in an overreached female population, and further investigation into the effects of oral contraceptives is completed before its use in practice.

## AUTHOR CONTRIBUTIONS

CB and JH conceived and designed the research. CB, JH, and GF performed the experimental laboratory procedures. All authors analyzed the data, interpreted the results, prepared the figures, and drafted and approved the manuscript final version.

## FUNDING INFORMATION

No funding was provided for this study.

## CONFLICT OF INTEREST STATEMENT

The authors declare no potential conflicts of interest, financial or otherwise.

## ETHICS STATEMENT

The study was approved by the Nottingham Trent University Invasive Ethics Committee (Ethics approval #573).

## Data Availability

The data that support the findings of this study are available from the corresponding author upon reasonable request.
